# The lived experiences of being physically active when morbidly obese: A qualitative systematic review

**DOI:** 10.3402/qhw.v10.28577

**Published:** 2015-09-22

**Authors:** Bente Skovsby Toft, Lisbeth Uhrenfeldt

**Affiliations:** 1 Department of Lifestyle Rehabilitation, Horsens Regional Hospital, Brædstrup, Denmark; 2 Department of Research, Horsens Regional Hospital, Denmark; 3 Department of Health, Science and Technology, Aalborg University, Aalborg, Denmark; 4 Danish Centre of Systematic Reviews: An Affiliate Centre of the Joanna Briggs Institute, Aalborg, Denmark

**Keywords:** Physical activity, morbid obesity, identity, self-efficacy, interpersonal relations, body image, qualitative research

## Abstract

The aim is to identify facilitators and barriers for physical activity (PA) experienced by morbidly obese adults in the Western world. Inactivity and a sedentary lifestyle have become a major challenge for health and well-being, particularly among persons with morbid obesity. Lifestyle changes may lead to long-term changes in activity level, if facilitators and barriers are approached in a holistic way by professionals. To develop lifestyle interventions, the perspective and experiences of this group of patients are essential for success. The methodology of the systematic review followed the seven-step procedure of the Joanna Briggs Institute and was published in a protocol. Six databases were searched using keywords and index terms. Manual searches were performed in reference lists and in cited citations up until March 2015. The selected studies underwent quality appraisal in the Joanna Briggs-Qualitative Assessment and Review Instrument. Data from primary studies were extracted and were subjected to a hermeneutic text interpretation and a data-driven coding in a five-step procedure focusing on meaning and constant targeted comparison through which they were categorized and subjected into a meta-synthesis. Eight papers were included for the systematic review, representing the experiences of PA among 212 participants. One main theme developed from the meta-data analysis: “Identity” with the three subthemes: “considering weight,” “being able to,” and “belonging with others.” The theme and subthemes were merged into a meta-synthesis: “Homecoming: a change in identity.” The experiences of either suffering or well-being during PA affected the identity of adults with morbid obesity either by challenging or motivating them. A change in identity may be needed to feel a sense of “homecoming” when active.

Inspired by the phenomenological lifeworld perspective of Husserl, a person's lifeworld is understood as a dynamic horizon in which we live and which “lives with us” (Husserl & Carr, 1970). The theory of the “lived body” by Merleau-Ponty (2002) has been the inspiration for exploring the first-person perspective of being in the world as a body. “It is never our objective body that we move, but our phenomenal body…” (p. 121). The body, which he considers the source of the lived experiences, is an integrated part of being in this world as both a subject and an object. In a phenomenological-hermeneutic approach, the lived experiences can be the starting point for identifying and influencing physical, mental, or logistical barriers to bodily movement such as physical exercise (Hills, Byrne, Lindstrom, & Hill, 2013). The accomplishment of changes can be facilitated or obstructed by experiences. The more barriers toward change that exist, the poorer the maintenance of the change accomplished (Elfhag & Rössner, 2005).

Galvin and Todres (2011) described a framework with six kinds of well-being and suffering in the model of a dwelling-mobility lattice. The framework was inspired by Heidegger's idea of mobility being movement in a nuanced way to move forward with time, space, and others, in a mood and with our bodies and a way to describe an access to the feeling of possibilities (Galvin & Todres, 2011). Human life and health are in this way balanced between suffering and well-being. Todres claims: “We live between suffering and well-being, just as we live between sky and earth” (Todres, Galvin, & Dahlberg, 2014, p. 114), and experiencing and working with both well-being and suffering is a part of all human life and human development. The metaphors of homeless and homecoming are inspired by Heidegger's ontological understanding of homecoming as well-being in our daily lives and the understanding of health as a way of being-at-home. The experience of facing existential homelessness is thought to provide mobility or motivation for change. To dwell is to come home to one's situation—feeling of homeness (Todres & Galvin, 2010).

The mobility of identity is based on the consideration that “I can.” It implicates self-efficacy, self-confidence, self-worth; positive and realistic dreams; and a feeling of capability in changes. Contrasting mobility, dwelling is another kind of well-being, which contains a possible feeling of acceptance, rootedness, and peace of identity when feeling well-being in “I am” (Galvin & Todres, 2011). This lattice has been the framework and inspiration for the analysis of findings in this review.

## People with large bodies

Referring to people with very large bodies is often done by classifying them as morbidly obese (MO). MO is commonly defined by the use of body mass index (BMI) ≥40 kg/m^2^ (or BMI 35–39.9 kg/m^2^ in combination with at least one weight-related comorbidity) (National Institute of Health and Clinical Excellence, 2006). In medical research, MO is considered a complex relapsing condition (Stubbs & Lavin, 2013) linked to several complications and limitations (Capodaglio et al., 2013; Hunskaar, 2008; McLaughlin & Hinyard, 2014; Wasserberg et al., 2008). Even with successful weight loss, weight is often regained over time in people with MO (Ohsiek & Williams, 2011; Wadden, Butryn, & Byrne, 2004). An inverse association between body weight and physical activity (PA) has been found (Kahn et al., 2002) with a very low prevalence of exercise among persons with MO as well as a reduced capacity to increase energy expenditure through PA (Stubbs & Lavin, 2013). Still, PA is considered the strongest health predictor (Kahn et al., 2002; Stubbs & Lavin, 2013) as strength and muscle mass can be preserved and weight gain/regain be prevented when people with MO increase their activity level (Hills & Byrne, 2004). In addition, PA is found to improve quality of life (Danielsen, Sundgot-Borgen, Mæhlum, & Svendsen, 2014; Jepsen et al., 2015), which is found to be particularly low among people with MO, and should be addressed specifically (McLaughlin & Hinyard, 2014). In addition, a strong predictor of mortality is being either MO and/or inactive (Flegal, Kit, Orpana, & Graubard, 2013; Gonzalez-Gross & Melendez, 2013; Hu et al., 2004; Kitahara et al., 2014).

## Systemworld or lifeworld as perspectives

In Habermas’ understanding, modern society is divided into two: a systemworld and a lifeworld. The systemworld, that is, the market economy and the state apparatus, and the lifeworld, that is, the world and culture in which man lives and human action takes place in a mutual understanding. The interaction of the formal, impersonal, and alienating systemworld and the values of the lifeworld are found to be important facilitating factors in a time dominated by the systemworld (Scheel, Pedersen, & Rosenkrands, 2008). In that way, people can be helped to find solutions compatible with their experiences of healthy living and self-care.

Inactivity and a sedentary lifestyle have become major challenges for health and well-being particularly among persons with MO. Lifestyle intervention can be understood from the point of view of both natural sciences and human and social sciences, which all should be approached and reflected on in a dynamic interaction to create a special knowledge and holistic understanding of human existence within health (Scheel et al., 2008). Lifestyle changes may lead to long-term changes in activity level if facilitators and barriers are approached in a holistic way by professionals. Practice should be founded on sciences to avoid interventions being based on health professionals’ own assumptions (Scheel et al., 2008). A broader holistic approach based on theoretical and evidence-based perspectives including well-being in those with obesity should be added (Brown & Wimpenny, 2011). Within this discourse of the systemworld, specifically in the context of a hospital setting, it seems important that health professionals do not miss the central importance of the person and their lifeworld (Galvin, 2010).

## Facilitators and barriers

A relationship between BMI and the number of barriers to PA has been identified (Cannioto, 2010), which could result in particularly difficult challenges for the people with MO (Zdziarski, Wasser, & Vincent, 2015). The perceived obesity stigma becomes more acute at higher weights (Lewis, Thomas, Blood, et al., 2011), and the feeling of being “too fat to exercise” is found to cause avoidance (Ball, Crawford, & Owen, 2000, p. 332; Vartanian & Shaprow, 2008) as well as a negative attitude toward PA (Piana et al., 2013). People with MO are found to experience more physical limitations (Hassan, Joshi, Madhavan, & Amonkar, 2003; Wang, Sereika, Styn, & Burke, 2013) and increased bodily pain symptoms during PA than others (Zdziarski et al., 2015), which may result in a lack of well-being during movements.

Facilitating positive experiences of PA is important when aiming for increased activity level. Particularly, the embodied experiences of people with large bodies, as the experiences might differ from people of smaller body sizes (Ball et al., 2000). It seems that well-being during PA can be developed among inactive persons, when small increases are made (Penedo & Dahn, 2005; Roqué i Figuls et al., 2013; Saris et al., 2003). Exploring health and well-being can reveal how illness, vulnerability, disability, and well-being might be intertwined, and how the person despite these factors finds a meaningful and a good life (Galvin et al., 2008).

### Aims

The aim was to identify the facilitators and barriers for PA experienced by MO adults in the Western world. The threefold research questions were:

1. How do adults with MO experience PA?
2. What facilitators for PA are experienced among adults with MO?
3. What barriers for PA are experienced among adults with MO?

## Design and methods

This study is designed as a qualitative meta-synthesis (The Joanna Briggs Institute, 2014) based on a systematic review. The target phenomenon was primarily identified in a clinical practice of lifestyle modification, which has inspired the research question, the definition of the Population, and the phenomenon of Interest and the Context (PICo) of the review. A search in the PROSPERO database (Clifford Neuman, 1992) showed no previous or present reviews on our topic. The protocol based on the Joanna Briggs Institute (JBI) procedure was published previously (Toft & Uhrenfeldt, 2014).

### Inclusion and exclusion criteria

Studies focusing on qualitative data on MO (BMI ≥40 kg/m^2^) adults (≥18 years) in the Western world, for example, the countries of Europe, Australia, and North America, were included. Also, studies investigated the experiences of facilitators for and barriers to PA, for example, research on the experiences of PA in recreational and leisure-time activity, transportation (e.g., walking or cycling), and occupational and household chores. Exclusion criteria were studies about mentally ill persons, pregnant women, children, and adolescents, and articles in languages other than English, German, Danish, Norwegian, or Swedish.

### Search methods

We initiated a three-step search method. Search terms based on the target of PICo elements were identified including MeSH terms and free text (Box 1). The search used all identified keywords and index terms that were systematically undertaken across the databases MEDLINE, CINAHL, and Embase. To validate the searches, we involved a research librarian in the search and retrieval processes.

**Box 1. Systematic search terms referring to PICo.**

Sedentary lifestyle OR sports OR activity* of daily living OR exercise* OR leisure activity* OR physical activity*

AND obese OR obesity OR bariatric*

AND perceived OR attitude* OR benefit* OR limitation* OR participation OR barriers OR facilitators OR motivate* OR experience* OR psychosocial factors OR physical factors

AND “Behavior and Behavior Mechanisms+” [MeSH]

Gray literature, for example, master's theses and doctoral dissertations, was searched for on Google Scholar, Mednar, and ProQuest.

**Figure 1:**
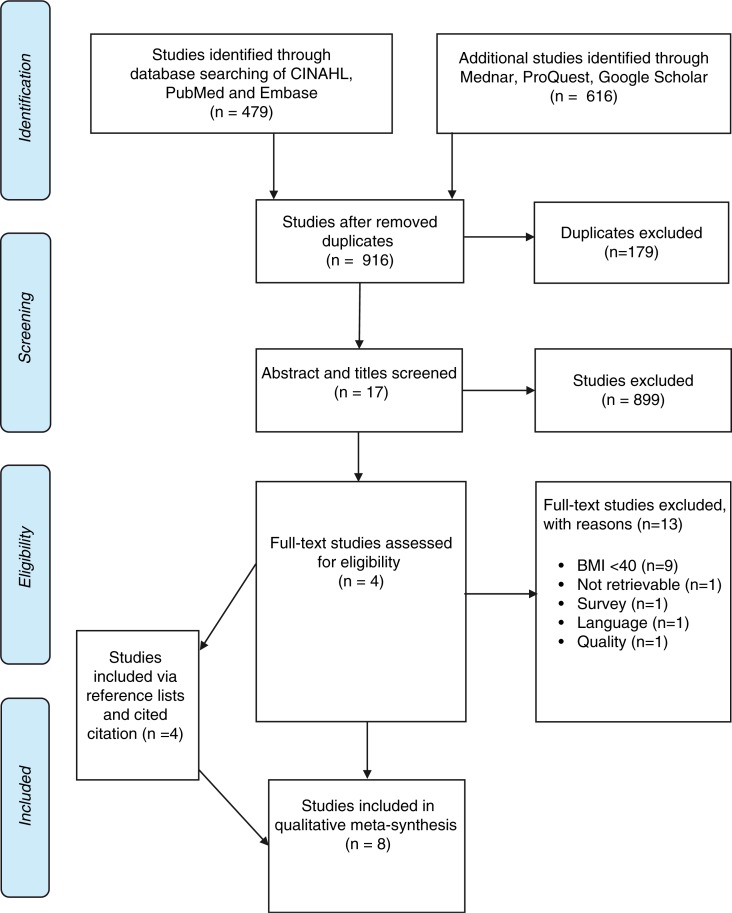
PRISMA flow chart of inclusion process (Moher, Liberati, Tetzlaff, & Altman, 2009).

The reference lists of all identified studies were searched for additional studies by both authors and citations, and a cited citation search on relevant studies was conducted (Figure 1). The searches were performed from March 2014 until March 2015.

For the first three facets, search terms including MeSH terms and free text were used. For the fourth facet, only the MeSH term in exploded form (+) was used.

### Search outcome

The electronic reference management program, RefWorks, was used to keep track of references and to remove duplicates. The searches resulted in a total of 916 studies after duplicates were removed. Title and abstracts were screened, and 17 full-text studies were assessed by both authors to match the review question and the inclusion criteria. In total, eight studies were included in the final review. Four studies were included by systematically searching the databases. One additional study was included via the search in reference lists and another three studies were identified in cited citations (Figure 1).

### Critical appraisal

The primary studies included underwent a critical appraisal conducted by both authors to evaluate the methodological validity of the studies (Hannes & Macaitis, 2012). Ten standardized questions were used in the web-based, JBI Qualitative Assessment and Review Instrument, and a four-point scale (yes, no, unclear, and not applicable) was applied (Pearson, Wiechula, Court, & Lockwood, 2005).

### Meta-data analysis

The meta-data analysis procedure was inspired by hermeneutic text interpretation for an inductive theory development based on a data-driven coding, and the findings were analysed by both authors.

For this review, a meta-aggregative approach was used by identifying findings, grouping the findings into subthemes, and synthesizing the subthemes into themes (The Joanna Briggs Institute, 2014, pp. 67–70). Data extraction from both quotations by participants (Sandelowski, 1994) and paraphrases by the authors of the primary studies were extracted using the JBI procedure for meta-synthesis (The Joanna Briggs Institute, 2014).

To provide structure and overview of the findings of the included articles, the five-step procedure described by Kvale and Brinkmann (2009) was used as a method of analysis using a procedure focusing on meaning: meaning condensation, meaning coding, and meaning interpretation. Meaning condensation was done by shortening the findings into condensed meaning units. Coding of the findings was done by attaching one or more keywords addressing different themes. Meaning interpretations beyond what was directly said were used to create a structure for themes and subthemes (Kvale and Brinkmann, 1996, 2009).

By searching for similarities and differences between the target phenomenon, subthemes and themes were integrated, as opposed to comparing them. Finally, an abstracted integration of findings was created via meta-synthesis inspired by the constant targeted comparison described by Sandelowski and Barroso (2007). This was used as an analytic device for the interpretative integration of qualitative findings in the primary research reports. These findings were taken as a whole and reduced to sets of abstracted statements before conclusions on the findings were drawn. The framework of Galvin and Todres (2011) was used for the meta-aggregation and meta-synthesis.

## Results

The review led to the inclusion of 8 papers, representing the experiences of 212 participants from five different countries: England, Australia, the United States, Sweden, and Norway. Most of the participants were women (*n*=143), and only 69 participants were men. One study only included men (Lewis, Thomas, Hyde, et al., 2011); two other studies only included women (Baruth, Sharpe, Parra-Medina, & Wilcox, 2014; Groven & Engelsrud, 2010). No homogenous criticism was found by the critical appraisal, but four of the eight studies had single individual issues that the reviewers agreed to comment upon (Baruth et al., 2014; Guess, 2012; Lewis, Thomas, Hyde, et al., 2011; Thomas, Hyde, Karunaratne, Kausman, & Komesaroff, 2008). An overview of the aim, sample, design method of analysis, and context of the primary studies (*n*=8) are illustrated in a meta-summary (Table I). Only findings on the experiences of participants with MO in each result sections were used in relation to the phenomenon of interest.

**Table I:** Meta-summary of included studies.

Author (year) country	Aim/objective	Sample (*n*=212) and design	Data analysis method	Context	Author's conclusions
Baruth (2014) USA	To explore perceptions and experiences with barriers to exercise and healthy eating among women from predominantly African American disadvantaged neighborhoods.	Females (*n*=28) males (*n*=0) Mean BMI: 40.4 kg/m^2^ (range 27.6–57.6) Mean age: 34.3 years (range 25–50.3) Open-ended, qualitative, focus-group interviews.	Transcripts, codes, and identification of key themes.	Disadvantaged neighborhoods, South Carolina.	Individual, social, and environmental factors were frequently mentioned as barriers to exercise and healthy eating. Insults from strangers about their body size and feelings of intimidation and embarrassment about not being able to complete exercises due to their body size were described as barriers to exercise.
Christiansen (2012) Norway	To gain more knowledge about characteristics of eating habits and body image as well as motivational forces for change.	Females (*n*=7) males (*n*=4) (9 of them awaiting bariatric surgery) BMI>40 kg/m^2^ (or>35 kg/m^2^ with one comorbidity) Age: range 26–56 years Open, narrative, qualitative interviews.	“Bricolage,” hermeneutic interpretation.	40-h weight reduction course in a patient education resource center.	Seeing oneself as an obese person was a gradual process that implied experiencing oneself as different from others. To experience a gap between knowing and doing concerning food habits in everyday life indicated that informants valued that they had a choice.
Dahl (2014) Norway	To describe how personnel argued for and perceived a residential weight-loss program, to investigate how the participants experienced the program, and to contrast these perspectives.	Females (*n*=5) males (*n*=3) BMI: range 40–63 kg/m^2^ Age: range 22–56 years. Observations, semi-structured focus-group interviews, and individual interviews.	Interview transcripts and field notes were inductive and thematically analysed together.	18-week on-site residential program at a weight-loss center in Denmark.	Participants embraced and adapted to the exercise part of the program. However, the personnel claimed that social training and personal development was necessary to lose weight, and weight to maintenance was not supported by all participants.
Lewis (2011) Australia	To investigate obese men's health behaviors and strategies for change.	Females (*n*=0) males (*n*=36) Mean BMI: 37.1 kg/m^2^ (range 30–60.7) Mean age: 45.5 years (range 21–69) Qualitative interviews by telephone.	Constant comparative methods of analysis. Transcripts, codes, and identification of themes.	Everyday life in community.	Men were found to feel a personal responsibility for weight gain. PA was seen as an empowering option, but difficult to fit into daily lives. Causes of weight gain and barriers to weight loss were sedentary lifestyles, stress, lack of work life balance, and weight-based stigma.
Groven (2010) Norway	To show how high-intensity training was experienced from a first-person perspective.	Females (*n*=5) males (*n*=0) Mean BMI: 44.8 kg/m^2^ (range 40–48) Age: range 35–63 years Face-to-face, semi-structured, in-depth interviews.	Kvale and Brinkman: transcribed, “bricolage,” meaning condensation; thematizing central themes, meaning interpretation.	Group-based treatment program.	Experiences of training were connected to the participants’ general experiences of being overweight. Both relationships to other people and earlier experiences were important for how the training was carried out.
Guess (2012) England	To examine the views and attitudes toward aerobic and resistance exercise among overweight and obese individuals.	Females (*n*=25) males (*n*=5) Mean BMI: 33.8 kg/m^2^ (SD=7.9) Mean age: 40.7 years Qualitative interviews and focus-group interviews and follow-up interviews.	Thematic analysis of transcribed data (NVIVO8).	12-week aerobic or resistance exercise program in a weight management clinic in London.	Weight loss was found as primary motivation for PA participation among women. Existing knowledge need to be reconciled to a successful weight loss and maintenance due to PA as participants had only little awareness or experience of resistance exercise and were fearful of potential risks.
Thomas (2008) Australia	To explore the extent to which people living with obesity have attempted to lose weight; their attitudes toward dieting, physical exercise and weight-loss solutions.	Females (*n*=63) males (*n*=13) Mean BMI: 42.5 kg/m^2^ (range 30–72,1) Mean age: 47 years (range 16–42) Open-ended, qualitative interviews face to face or by telephone.	Constant continuous, comparative method.	Urban and rural areas in Victoria, Australia.	Very few individuals with obesity were given appropriate long-term guidance or support. The positive role of social networks seemed particularly important in engaging individuals in PA.
Wiklund (2011) Sweden	To describe how adults with severe obesity, awaiting gastric bypass surgery experience PA.	Females (*n*=10) males (*n*=8) Mean BMI: 47 kg/m^2^ (range 29–62) Mean age: 47 (range 18–65) In-depth, semi-structured interviews.	7 Steps of Dahlgren and Fallsberg.	Hospital and home setting. Awaiting gastric bypass surgery.	PA was experienced positively, but obstacles exist that influence the capacity or the will of the people living with severe obesity. Support is necessary to maintain PA.

### Meta-synthesis: “Homecoming: a change in identity”

The meta-synthesis was developed from the theme and subthemes. The meaning of homecoming is to be understood as becoming comfortable and content in body, heart, and head, aiming for an energized and vitalized change or development of the identity.

“Identity” was found as a central theme for PA among MO adults and developed from three subthemes: “considering weight,” “being able to,” and “belonging with others” (Table II). The subthemes are to be understood as being homeless when not feeling at home in their present body and/or situation.

**Table II:** Coherence between meta-synthesis, theme, and subthemes in the review.

Meta-synthesis		Homecoming: a change in identity	
Main theme		Identity—a sense of self	
Subthemes	Considering weight	Being able to	Belonging with others

### Identity

The identity of the individuals in the studies was addressed in all subthemes according to the idea of an ideal body size relating to their own sense of self which was affecting the terms of change. The identity of the participants was influenced by mood (e.g., motivation, enjoyment, fear, and embarrassment) as well as embodiment (e.g., appearance, limitations, performance, and accessibility), spatiality (e.g., environment and costs), temporality (e.g., time and obligations), and intersubjectivity (e.g., network, support, and guidance).

### Considering weight

In all the included studies, participants’ changes in identity were affected by considering weight. Both experiences of weight loss and weight gain were affecting the identity of the participants. Being weighed was found necessary to stay on track (Groven & Engelsrud, 2010), but was also mentioned as “being stressful” (Groven & Engelsrud, 2010, p. 5133). A person said, “to be measured and have one's weight checked, and be told you have fault was no good” (Christiansen, Borge, & Fagermoen, 2012, p. 17259). This experience illustrated that the procedure for being weighed could for some result in what we interpret as being homeless in one's own identity.

Weight loss was found to be not only difficult but also an important aim in achieving ideal body size, freedom (Groven & Engelsrud, 2010), possibilities, and becoming attractive (Thomas et al., 2008), as well as gaining mobility. A person reasoned that “My body hurts, especially my knees. But I think this has to do with me being too heavy. So I really need to lose more weight” (Groven & Engelsrud, 2010, p. 17259).

Weight loss was for different reasons found interrelated with PA and was considered the primary motivation and reason for exercising (Dahl, Rise Kulseng, & Steinsbekk, 2014; Groven & Engelsrud, 2010; Guess, 2012; Lewis, Thomas, Hyde, et al., 2011; Thomas et al., 2008; Wiklund, Olsen, & Willen, 2011).

A 24-year-old female stated:

I mean, does the person really come to the doctor and say I want to exercise because I want to be healthy or send me to the gym because I want to be healthy? No! They go on exercise and diet programs because they want to lose weight. (Guess, 2012, p. 195)

In this quote, weight loss was linking diet and exercise together, rather than health, as the driving force in the mobility toward a reach for homecoming.

Therefore, lack of weight loss, too slow a weight loss, or reducing certain parts of their body by increased PA influenced their attitude toward exercise negatively and prohibited the feeling of homecoming. One person said that, “You can't stay committed [to exercise] because you don't see the results” (Baruth et al., 2014, p. 342). This finding illustrated that exercise seems to be determined by weight loss and PA was reduced to being a weight control tool.

In all the included studies, weight gain was mainly considered a negative experience linked to feeling guilty (Groven & Engelsrud, 2010) and dissatisfaction (Thomas et al., 2008). Different statements showed that weight gain was considered either a negative result of PA or a negative result of inactivity in both cases adding a kind of homelessness to the situation. One female participant said:

If you exercise … you might not lose anything. You might actually gain it because you might be gaining muscle … you think, Oh my god I haven't lost anything, I've gained, so then you might give up after that … and you just think you know. I can't do this, I can't be bothered. (Guess, 2012, p. 195)

Contrasting this experience, another person experienced bodily well-being, “unfortunately, I didn't lose weight. But it was nice to sense your own muscles. And I really enjoyed the slow movements. It was really nice” (Groven & Engelsrud, 2010, p. 5132).

This good experience of movement is one of the considerations of being active regardless of weight loss. However, most considerations represented homelessness in the sense that the exercise supposed to make you lose weight did not.

In one study, weight gain was considered positive and large curves regarded as an ideal (Baruth et al., 2014). No one mentioned weight gain as a positive result of PA or exercise and no one mentioned positive physiological adaptations to PA which might be achieved. In conclusion, lack of weight loss or weight gain affected the motivation and activity level negatively.

### Being able to

The second subtheme identified was the participants’ experiences of being able to or thinking “I can.” The positive experiences were related to success in achieving weight loss, or improved skills (Dahl et al., 2014), and were facilitating changes. Some found intensions and actions divergent (Baruth et al., 2014) and considered themselves unable to use their knowledge (Christiansen et al., 2012; Groven & Engelsrud, 2010).

A person stated, “Give me a personal trainer that gets me out of bed every morning and makes me exercise, and yeah, I'd lose weight” (Thomas et al., 2008, p. 5). This quote could indicate that the person was feeling unable to generate changes on his own.

Health benefits of PA were mentioned by a female (24 years), “Risk of diabetes. Heart disease, I think it can work toward combating a lot of those things” (Guess, 2012, p. 195). The risk of diseases was in this case the motivation for changes.

The experiences of choosing to exercise alone were also mentioned in relation to attaining an individually adjusted activity level in pace, intensity, or durance (Wiklund et al., 2011). One person experienced that, “The instructors drove you to exhaustion or it felt like you didn't do anything … either it was too fast or too slow” (Wiklund et al., 2011, p. 183) and another person explained, that:

If I'm out walking with someone, for example my friends, they usually walk faster than I do. I try to keep up but I don't have the energy. Then maybe I take a walk by myself instead at my own pace. (p. 183)

This person and a few others (Guess, 2012; Wiklund et al., 2011) expressed reduced physical performance in exercise; however, they perceived self-management and an individual pace without pressure as positive. The quote showed homelessness in mobility with others, but reaching for homecoming by choosing to walk alone.

The experiences of trust (Baruth et al., 2014), enjoyment (Baruth et al., 2014; Wiklund et al., 2011), and comfort in the activities; realistic expectations in achieving goals and avoiding disappointments (Baruth et al., 2014; Dahl et al., 2014; Groven & Engelsrud, 2010; Thomas et al., 2008; Wiklund et al., 2011); as well as feeling safe (Baruth et al., 2014; Christiansen et al., 2012) were all found to be both facilitators and inhibitors for changes in different persons. Pressure was mainly experienced as negative (Wiklund et al., 2011), but positive pressure from a health professional was considered supportive (Groven & Engelsrud, 2010). In general, individual support from authentic professionals was warranted (Dahl et al., 2014; Groven & Engelsrud, 2010; Thomas et al., 2008), as it created an enjoyable, safe atmosphere (Baruth et al., 2014; Christiansen et al., 2012; Dahl et al., 2014). Individual guidance and realistic plans (Thomas et al., 2008) and advice and follow-up in lifestyle modifications (Dahl et al., 2014; Groven & Engelsrud, 2010; Wiklund et al., 2011) were considered helpful in relation to being able to exercise. A person participating in a residential weight-loss program told about the experience of being met with support and respect, “that is how they [the staff] build our self-confidence. We are all of a sudden humans again and not just the chubby clown” (Dahl et al., 2014, p. 100229). Experiences of such staff may enhance the identity or the sense of self among the participants moving from homeless to a kind of homecoming in an individually perceived identity.

However, some had the experience of having their problems generalized and predicted by staff, which was not supportive. The participants would prefer being met respectfully and without prejudice (Dahl et al., 2014).

In general, more bad than good experiences from not “being able to” were mentioned. The bad experiences of physical health problems and weight (Christiansen et al., 2012; Thomas et al., 2008) or limitations due to injury (Baruth et al., 2014; Guess, 2012), body size, pain, and discomfort (Groven & Engelsrud, 2010) were all found to be obstacles to PA and as such a kind of homelessness. One participant explained:

My head knew what I was supposed to do, but my legs just wouldn't respond. It just didn't work; I couldn't keep up, with the knees and everything. It didn't work at all and that's so awfully frustrating when you want to do something you can't that you've done for years. (Wiklund et al., 2011, p. 182)

This quote illustrates the suffering in identity which may lead to the feeling of homelessness within the embodied possibilities.

The capability of exercising was negatively influenced by the considerations of one's appearance, and worries whether exercise equipment would be proportioned to large bodies. The feelings of embarrassment (Thomas et al., 2008), humiliation, intimidation (Baruth et al., 2014), failure (Groven & Engelsrud, 2010), and self-blame resulted in feeling homeless during PA. All these experiences were linked to the identity or body image of the person. In addition, the individual's priority and motivation (Baruth et al., 2014), their preferences and attention (Christiansen et al., 2012), as well as initiative and commitment (Thomas et al., 2008) were explicitly mentioned as hindering them from being able to increase their activity level.

Finally, practical circumstances (Thomas et al., 2008), costs, time (Baruth et al., 2014; Thomas et al., 2008), and lack of safety were experienced as causing the diversion between intension and action (Baruth et al., 2014) and blocking development and change.

### Belonging with others

The third subtheme “belonging with others” was developed from the influence of intersubjectivity on the experiences of PA as well as eating habits and weight loss (Baruth et al., 2014; Thomas et al., 2008).

Most of the experiences identified related to the feeling of being homeless in PA among others within the different contexts. The experiences mentioned were mainly perceived as uncomfortable and a lack of well-being.

A man, aged 55, told that, “Just walking into a gymnasium is hugely embarrassing. You may as well walk in there naked because everyone turns to you and looks at you and you can just about hear them going ‘oh yuck’”(Lewis, Thomas, Hyde, et al., 2011, p. 465). The experience in this quote is related to stigma and bodily disgrace, inhibiting the ambitions of change toward homecoming in body and movement.

The good experiences were linked to experiences of good mood, being comfortable, being able to enjoy, feeling safe, and being encouraged (Baruth et al., 2014; Christiansen et al., 2012; Dahl et al., 2014; Groven & Engelsrud, 2010; Wiklund et al., 2011) imbibing the community spirit in the company of others (Groven & Engelsrud, 2010; Guess, 2012; Thomas et al., 2008). A person experienced that, “Exercising in a pool is lots of fun and especially if it's a fun group, then it's a social activity at the same time… really great to have someone to exercise with” (Wiklund et al., 2011, p. 184).

Network and an exercise partner were considered helpful, but particularly exercising with peers (Dahl et al., 2014; Groven & Engelsrud, 2010) in a treatment context was perceived as a positive obligation (Wiklund et al., 2011). A supportive network (Dahl et al., 2014; Thomas et al., 2008) was facilitating recognition, acceptance, and the sharing of problems (Groven & Engelsrud, 2010). PA was found to both enhance isolation and socialization (Thomas et al., 2008) depending on the experiences of the individual, and identity was influenced by embodiment, intersubjectivity, and mood. A woman said: “I do not feel ashamed of my body here. We are all in the same situation, you see, which is really nice” (Groven & Engelsrud, 2010, p. 5131). This quote illustrates how the sense of belonging with peers felt like a kind of homecoming. Homecoming was found in increased respect by others (Dahl et al., 2014), motivation, self-confidence, and self-worth (Dahl et al., 2014). Participating in programed activities was found to increase self-insight (Dahl et al., 2014).

In all studies, participation in a group context or in public was also associated with the opposite feelings due to conflicts, ashamedness, modesty, or fear of being ridiculed.

A female, 45 years old, said that, “I would rather work indoors, put some music on and work out myself. Cause I am too self-conscious to go to the gym with all these skinny little women” (Guess, 2012, p. 198) and a man stated that, “Doing any physical activity, it's not something to look forward to, it's putting myself out there to be ridiculed again” (Lewis, Thomas, Hyde, et al., 2011, p. 464). Both felt a lack of belonging and homelessness with others.

Some participants felt they had to endure pain and uncomfortable challenges (Baruth et al., 2014; Groven & Engelsrud, 2010; Wiklund et al., 2011) due to pressure or advice from strangers, family, friends, or professionals (Baruth et al., 2014; Groven & Engelsrud, 2010; Thomas et al., 2008; Wiklund et al., 2011). Others were particularly sensitive to reactions such as being looked at (Christiansen et al., 2012) or getting negative comments (Thomas et al., 2008), which were not supportive for the continuance of new habits.

Societal factors such as unsafe environment, that is, traffic and darkness were regarded constraints for exercise (Baruth et al., 2014). Healthy living was influenced by family customs and traditions (Baruth et al., 2014; Christiansen et al., 2012; Groven & Engelsrud, 2010), different commitments, and responsibilities in the family (Baruth et al., 2014; Guess, 2012). In addition, the priority of time and money was found to be a barrier to exercise (Baruth et al., 2014; Thomas et al., 2008).

## Discussion

A whole palette of experiences of suffering and well-being and being a “big size body” in activity was found to facilitate or inhibit PA. The meta-synthesis of this study added the knowledge of how facilitators and barriers, in an existential dynamic way, can make one feel at home or homeless in an activity. Reducing suffering and increasing well-being were found to involve a change in identity. A previous article has been conducted on doing, being, becoming, and belonging as dimensions affecting the experiences of PA. The concepts of becoming and belonging were considered underdeveloped, but were found relevant analysing and addressing, to add greater depth to the understanding of peoples’ experiences (Hitch, Pépin, & Stagnitti, 2014). The meta-synthesis of this review supports this thinking of a holistic way to approach good practice and establishing a shared language.

A barrier to homecoming was lack of language (Warin & Gunson, 2013) to address this group of people with dignity, which was found problematic. It has been considered important to avoid unattractive assumptions in this research on obesity, but as predicted (Burkhauser & Cawley, 2008), alternate specifications other than MO has been hard to find. In future research, we would select “severe” rather than “morbid,” to add an understanding which represents the burden on the person rather than a risk factor from the systemworld.

A limitation of the study might be the use of BMI obesity class III (BMI≥40 kg/m^2^) defined by WHO (World Health Organization, 2004). It was chosen as inclusion criteria for the process of the structured, systematic, literature search, even though the classification by some has been considered outdated (De Lorenzo et al., 2013). It was deliberately chosen to identify only the experiences of the most obese participants. Still, it turned out to be problematic to identify this specific population within the studies screened, as there seemed to be no tradition for distinguishing between the most obese and the less obese. For that reason, offhand interesting articles, which did not concern persons with MO, had to be excluded from this review (Bove & Olson, 2006; Chang, Nitzke, Guilford, Adair, & Hazard, 2008; Peacock, Sloan, & Cripps, 2014; Piana et al., 2013).

Because of the results of this study, we suggest addressing the experiences of well-being, the capability of doing, and the dimension of belonging with others when intervening. The need for change in identity in the process from homeless to homecoming might add concepts that can be understood and shared within different professions in a lifeworld-led language of dignity that will help facilitate change.

This review supports the idea that a successful health intervention implicates both professional guidance and patients’ empowerment in achieving control of their own lives (Kitson, Harvey, & McCormack, 1998, p. 115), and it might help the professionals to identify feasible interventions (Dalle Grave et al., 2010) by taking the patient's perspective into account (Piana et al., 2013) by uncovering their lived experiences.

Multiple barriers to PA have been identified and the main theme “identity” has previously been highlighted in a review of qualitative studies, as participating in sports and PA was found to be a challenge to identity in having to show others an unfit body (Allender, Cowburn, & Foster, 2006). This was supported in another review on psychological factors influencing weight-loss maintenance, where it was found that a positive body image was an important factor for success (Ohsiek & Williams, 2011). Multiple experiences seem to become more negative with increased weight and the sense of self may be affected differently, as BMI seems to correlate with perceived barriers and self-efficacy (Stutts, 2002). This indicates the importance of intervention among persons with MO with relevant and realistic personal plans to increase the feeling of homecoming when physically active. Particularly, positive experiences in relation to adjusted activities, positive atmosphere with others, and professional support with both “head, hand and heart” (Galvin, 2010, p. 170) were associated with well-being. Activities with others can both cause isolation and socialization, and stigma can be a barrier to PA.

## Implication for practice

Professionals and leaders may use the meta-synthesis to help address suffering and well-being among adults living with MO in practice. A lifeworld-led PA approach focusing on embodied experiences, recognizing thoughts and feelings in the present situation, may be helpful when addressing the lifestyle of the individuals with respect to their values. In group interventions promoting PA, the relations and kinship with peers as well as the environmental setting seem most relevant to address.

In healthcare practice, the results of this meta-synthesis may lead to an evidence-based, authentic, and humanistic approach of the lived experiences which may enhance healthcare professionals’ respect and dignity toward patients (Ludvigsen et al., 2015). Future research may focus on how to make a successful meeting of lifeworld and systemworld from an existential point of view, due to the need for achieving long-term outcome of PA interventions among people with MO.

## Conclusions

Adults with MO were found to have multiple challenges when being active. The meta-synthesis developed was: “Homecoming: a change in identity,” referring to the experiences of either suffering or well-being during PA among people with large bodies. To make a change in identity, different facilitators and barriers were found to affect their sense of self by “considering weight,” “being able to,” and/or “belonging with others,” themes which were found relevant to approach when aiming for an increased activity level among persons with MO. This review has added scientific and systematic knowledge on the experiences of identity within the lived body and of lived relations and the challenges of being physically active when living with MO.

## References

[CIT0001] Allender S, Cowburn G, Foster C (2006). Understanding participation in sport and physical activity among children and adults: A review of qualitative studies. Health Education Research.

[CIT0002] Ball K, Crawford D, Owen N (2000). Too fat to exercise? Obesity as a barrier to physical activity. Australian and New Zealand Journal of Public Health.

[CIT0003] Baruth M, Sharpe P. A, Parra-Medina D, Wilcox S (2014). Perceived barriers to exercise and healthy eating among women from disadvantaged neighborhoods: Results from a focus groups assessment. Women & Health.

[CIT0004] Bove C. F, Olson C. M (2006). Obesity in low-income rural women: Qualitative insights about physical activity and eating patterns. Women & Health.

[CIT0005] Brown J, Wimpenny P (2011). Developing a holistic approach to obesity management. International Journal of Nursing Practice.

[CIT0006] Burkhauser R. V, Cawley J (2008). Beyond BMI: The value of more accurate measures of fatness and obesity in social science research. Journal of Health Economics.

[CIT0007] Cannioto R. A (2010). Physical activity barriers, behaviors, and beliefs of overweight and obese working women: A preliminary analysis. Women in Sport & Physical Activity Journal.

[CIT0008] Capodaglio P, Lafortuna C, Petroni M. L, Salvadori A, Gondoni L, Castelnuovo G (2013). Rationale for hospital-based rehabilitation in obesity with comorbidities. European Journal of Physical and Rehabilitation Medicine.

[CIT0009] Chang M, Nitzke S, Guilford E, Adair C. H, Hazard D. L (2008). Motivators and barriers to healthful eating and physical activity among low-income overweight and obese mothers. Journal of the American Dietetic Association.

[CIT0010] Christiansen B, Borge L, Fagermoen M. S (2012). Understanding everyday life of morbidly obese adults-habits and body image. International Journal of Qualitative Studies on Health and Well-being.

[CIT0011] Clifford Neuman B (1992). Prospero: A tool for organizing internet resources. Internet Research.

[CIT0012] Dahl U, Rise M. B, Kulseng B, Steinsbekk A (2014). Personnel and participant experiences of a residential weight-loss program. A qualitative study. PLoS One.

[CIT0013] Dalle Grave R, Calugi S, Centis E, Marzocchi R, El Ghoch M, Marchesini G (2010). Lifestyle modification in the management of the metabolic syndrome: Achievements and challenges. Diabetes, Metabolic Syndrome and Obesity: Targets and Therapy.

[CIT0014] Danielsen K. K, Sundgot-Borgen J, Mæhlum S, Svendsen M (2014). Beyond weight reduction: Improvements in quality of life after an intensive lifestyle intervention in subjects with severe obesity. Annals of Medicine.

[CIT0015] De Lorenzo A, Bianchi A, Maroni P, Iannarelli A, Di Daniele N, Iacopino L (2013). Adiposity rather than BMI determines metabolic risk. International Journal of Cardiology.

[CIT0016] Elfhag K, Rössner S (2005). Who succeeds in maintaining weight loss? A conceptual review of factors associated with weight loss maintenance and weight regain. Obesity Reviews.

[CIT0017] Flegal K. M, Kit B. K, Orpana H, Graubard B. I (2013). Association of all-cause mortality with overweight and obesity using standard body mass index categories: A systematic review and meta-analysis. JAMA.

[CIT0018] Galvin K, Emami A, Dahlberg K, Bach S, Ekebergh M, Rosser E (2008). Challenges for future caring science research: A response to Hallberg (2006). International Journal of Nursing Studies.

[CIT0019] Galvin K. T (2010). Revisiting caring science: Some integrative ideas for the ‘head, hand and heart’ of critical care nursing practice. Nursing in Critical Care.

[CIT0020] Galvin K. T, Todres L (2011). Kinds of well-being: A conceptual framework that provides direction for caring. International Journal of Qualitative Studies on Health and Well-being.

[CIT0021] Gonzalez-Gross M, Melendez A (2013). Sedentarism, active lifestyle and sport: Impact on health and obesity prevention. Nutricion Hospitalaria.

[CIT0022] Groven K. S, Engelsrud G (2010). Dilemmas in the process of weight reduction: Exploring how women experience training as a means of losing weight. International Journal of Qualitative Studies on Health and Well-being.

[CIT0023] Guess N (2012). A qualitative investigation of attitudes towards aerobic and resistance exercise amongst overweight and obese individuals. BMC Research Notes.

[CIT0024] Hannes K, Macaitis K (2012). A move to more systematic and transparent approaches in qualitative evidence synthesis: Update on a review of published papers. Qualitative Research.

[CIT0025] Hassan M. K, Joshi A. V, Madhavan S. S, Amonkar M. M (2003). Obesity and health-related quality of life: A cross-sectional analysis of the US population. International Journal of Obesity and Related Metabolic Disorders: Journal of the International Association for the Study of Obesity.

[CIT0026] Hills A. P, Byrne N. M (2004). Physical activity in the management of obesity. Clinics in Dermatology.

[CIT0027] Hills A. P, Byrne N. M, Lindstrom R, Hill J. O (2013). Small changes to diet and physical activity behaviors for weight management. Obesity Facts.

[CIT0028] Hitch D, Pépin G, Stagnitti K (2014). In the footsteps of Wilcock, part one: The evolution of doing, being, becoming, and belonging. Occupational Therapy in Health Care.

[CIT0029] Hu F. B, Willett W. C, Li T, Stampfer M. J, Colditz G. A, Manson J. E (2004). Adiposity as compared with physical activity in predicting mortality among women. New England Journal of Medicine.

[CIT0030] Hunskaar S (2008). A systematic review of overweight and obesity as risk factors and targets for clinical intervention for urinary incontinence in women. Neurourology and Urodynamics.

[CIT0031] Husserl E, Carr D (1970). The crisis of European sciences and transcendental phenomenology: An introduction to phenomenological philosophy.

[CIT0032] Jepsen R, Aadland E, Robertson L, Kolotkin R. L, Andersen J. R, Natvig G. K (2015). Physical activity and quality of life in severely obese adults during a two-year lifestyle intervention programme. Journal of Obesity.

[CIT0033] Kahn E. B, Ramsey L. T, Brownson R. C, Heath G. W, Howze E. H, Powell K. E (2002). The effectiveness of interventions to increase physical activity: A systematic review. American Journal of Preventive Medicine.

[CIT0034] Kitahara C. M, Flint A. J, de Gonzalez A. B, Bernstein L, Brotzman M, MacInnis R. J (2014). Association between class III obesity (BMI of 40–59 kg/m^2^) and mortality: A pooled analysis of 20 prospective studies. PLoS Med.

[CIT0035] Kitson A, Harvey G, McCormack B (1998). Enabling the implementation of evidence based practice: A conceptual framework. Quality in Health Care.

[CIT0036] Kvale S, Brinkmann S (1996). Interviews: An introduction to qualitative research interviewing.

[CIT0037] Kvale S, Brinkmann S (2009). Interviews: Learning the craft of qualitative research interviewing.

[CIT0038] Lewis S, Thomas S. L, Blood R. W, Castle D. J, Hyde J, Komesaroff P. A (2011). How do obese individuals perceive and respond to the different types of obesity stigma that they encounter in their daily lives? A qualitative study. Social Science & Medicine.

[CIT0039] Lewis S, Thomas S. L, Hyde J, Castle D. J, Komesaroff P. A (2011). A qualitative investigation of obese men's experiences with their weight. American Journal of Health Behavior.

[CIT0040] Ludvigsen M. S, Hall E. O, Meyer G, Fegran L, Aagaard H, Uhrenfeldt L (2015). Using Sandelowski and Barroso's metasynthesis method in advancing qualitative evidence. Qualitative Health Research.

[CIT0041] McLaughlin L, Hinyard L. J (2014). The relationship between health-related quality of life and body mass index. Western Journal of Nursing Research.

[CIT0042] Merleau-Ponty M (2002). Phenomenology of perception.

[CIT0043] Moher D, Liberati A, Tetzlaff J, Altman D. G (2009). Preferred reporting items for systematic reviews and meta-analyses: The PRISMA statement. Annals of Internal Medicine.

[CIT0044] National Institute of Health and Clinical Excellence (2006). Obesity: The prevention, identification, assessment and management of overweight and obesity in adults and children.

[CIT0045] Ohsiek S, Williams M (2011). Psychological factors influencing weight loss maintenance: An integrative literature review. Journal of the American Academy of Nurse Practitioners.

[CIT0046] Peacock J. C, Sloan S. S, Cripps B (2014). A qualitative analysis of bariatric patients’ post-surgical barriers to exercise. Obesity Surgery.

[CIT0047] Pearson A, Wiechula R, Court A, Lockwood C (2005). The JBI model of evidence based Healthcare. International Journal of Evidence Based Healthcare.

[CIT0048] Penedo F. J, Dahn J. R (2005). Exercise and well-being: A review of mental and physical health benefits associated with physical activity. Current Opinion in Psychiatry.

[CIT0049] Piana N, Battistini D, Urbani L, Romani G, Fatone C, Pazzagli C (2013). Multidisciplinary lifestyle intervention in the obese: Its impact on patients’ perception of the disease, food and physical exercise. Nutrition, Metabolism, and Cardiovascular Diseases: NMCD.

[CIT0050] Roqué i Figuls M, Martínez García L, Martinez Zapata M. J, Pacheco R, Mauricio D, Cosp B (2013). Interventions for treating overweight or obesity in adults: An overview of systematic reviews. The Cochrane Library.

[CIT0051] Sandelowski M (1994). Focus on qualitative methods. The use of quotes in qualitative research. Research in Nursing & Health.

[CIT0052] Sandelowski M, Barroso J (2007). Handbook for synthesizing qualitative research.

[CIT0053] Saris W. H, Blair S. N, Van Baak M. A, Eaton S. B, Davies P. S, Di Pietro L (2003). How much physical activity is enough to prevent unhealthy weight gain? Outcome of the IASO 1st stock conference and consensus statement. Obesity Reviews: An Official Journal of the International Association for the Study of Obesity.

[CIT0054] Scheel M. E, Pedersen B. D, Rosenkrands V (2008). Interactional nursing—A practice theory in the dynamic field between the natural, human and social sciences. Scandinavian Journal of Caring Sciences.

[CIT0055] Stubbs R. J, Lavin J. H (2013). The challenges of implementing behaviour changes that lead to sustained weight management. Nutrition Bulletin.

[CIT0056] Stutts W. C (2002). Physical activity determinants in adults. Perceived benefits, barriers, and self efficacy. AAOHN Journal: Official Journal of the American Association of Occupational Health Nurses.

[CIT0057] The Joanna Briggs Institute (2014). Joanna Briggs Institute Reviewers’ Manual: 2014 edition.

[CIT0058] Thomas S. L, Hyde J, Karunaratne A, Kausman R, Komesaroff P. A (2008). They all work … when you stick to them: A qualitative investigation of dieting, weight loss, and physical exercise in obese individuals. Nutrition Journal.

[CIT0059] Todres L, Galvin K (2010). “Dwelling-mobility”: An existential theory of well-being. International Journal of Qualitative Studies on Health and Well-being.

[CIT0060] Todres L, Galvin K. T, Dahlberg K (2014). Caring for insiderness: Phenomenologically informed insights that can guide practice. International Journal of Qualitative Studies on Health and Well-being.

[CIT0061] Toft B. S, Uhrenfeldt L (2014). Facilitators and barriers to physical activity experienced among morbidly obese adults: A systematic review protocol. JBI Database of Systematic Reviews & Implementation Reports.

[CIT0062] Vartanian L. R, Shaprow J. G (2008). Effects of weight stigma on exercise motivation and behavior: A preliminary investigation among college-aged females. Journal of Health Psychology.

[CIT0063] Wadden T. A, Butryn M. L, Byrne K. J (2004). Efficacy of lifestyle modification for long term weight control. Obesity Research.

[CIT0064] Wang J, Sereika S. M, Styn M. A, Burke L. E (2013). Factors associated with health-related quality of life among overweight or obese adults. Journal of Clinical Nursing.

[CIT0065] Warin M. J, Gunson J. S (2013). The weight of the word: Knowing silences in obesity research. Qualitative Health Research.

[CIT0066] Wasserberg N, Haney M, Petrone P, Crookes P, Rosca J, Ritter M (2008). Fecal incontinence among morbid obese women seeking for weight loss surgery: An underappreciated association with adverse impact on quality of life. International Journal of Colorectal Disease.

[CIT0067] Wiklund M, Olsen M. F, Willen C (2011). Physical activity as viewed by adults with severe obesity, awaiting gastric bypass surgery. Physiotherapy Research International: The Journal for Researchers and Clinicians in Physical Therapy.

[CIT0068] World Health Organization (2004). BMI classification.

[CIT0069] Zdziarski L. A, Wasser J. G, Vincent H. K (2015). Chronic pain management in the obese patient: A focused review of key challenges and potential exercise solutions. Journal of Pain Research.

